# The Role of Oxidative Stress in the Pathomechanism of Congenital Malformations

**DOI:** 10.1155/2018/7404082

**Published:** 2018-12-30

**Authors:** Nicola Laforgia, Antonio Di Mauro, Giovanna Favia Guarnieri, Dora Varvara, Lucrezia De Cosmo, Raffaella Panza, Manuela Capozza, Maria Elisabetta Baldassarre, Nicoletta Resta

**Affiliations:** ^1^Neonatology and Neonatal Intensive Care Unit, Department of Biomedical Science and Human Oncology, “Aldo Moro” University of Bari, Policlinico Hospital-Piazza Giulio Cesare n. 11, 70124 Bari, Italy; ^2^Medical Genetics Unit, Department of Biomedical Sciences and Human Oncology, “Aldo Moro” University of Bari, Policlinico Hospital-Piazza Giulio Cesare n. 11, 70124 Bari, Italy

## Abstract

Congenital anomalies are significant causes of mortality and morbidity in infancy and childhood. Embryogenesis requires specific signaling pathways to regulate cell proliferation and differentiation. These signaling pathways are sensitive to endogenous and exogenous agents able to produce several structural changes of the developing fetus. Oxidative stress, due to an imbalance between the production of reactive oxygen species and antioxidant defenses, disrupts signaling pathways with a causative role in birth defects. This review provides a basis for understanding the role of oxidative stress in the pathomechanism of congenital malformations, discussing the mechanisms related to some congenital malformations. New insights in the knowledge of pathomechanism of oxidative stress-related congenital malformations, according to experimental and human studies, represent the basis of possible clinical applications in screening, prevention, and therapies.

## 1. Introduction

Embryogenesis represents a complex process requiring temporal and spatial regulatory mechanisms [1]. These mechanisms have evolved to be particularly resilient to stressor factors, but experimental studies have shown that embryonic stages are very sensitive to internal or external stressors because of reduced protecting mechanisms [2].

Environmental drugs, chemicals, and physical agents can produce congenital malformations and reproductive effects. The most common known cause is genetic, but the largest group, unfortunately, is unknown.

It is important to remember that a teratogenic exposure includes not only the agent but also the dose and the time in pregnancy when the exposure has to occur. The dose is a crucial component in determining the risk, since those teratogenic agents follow a toxicologic dose-response curve [3].

Known agents that have been demonstrated to result in malformations cannot produce every type of malformation. So, it is easier to exclude an agent as a cause of birth defects than to conclude definitively that it was responsible for birth defects [3].

Oxidation–reduction (redox) homeostasis, like pH control, is central to life. Redox processes pervade practically all fundamental processes of life from bioenergetics to metabolism and life functions [4]. Biological redox reactions are manifold and organized according to the principles of the redox code [5].

Oxidative stress is an imbalance between oxidants and antioxidants in favour of the oxidants, leading to a disruption of redox signaling and control and/or molecular damage [6]. Oxidative stress is two sided; whereas excessive oxidant challenge causes damage to biomolecules, maintenance of a physiological level of oxidant challenge, termed oxidative eustress, is essential for governing life processes through redox signaling [4].

Biological redox equilibria do not denote, as a matter of fact, true thermodynamic equilibria but instead are “nonequilibria” as defined by steady state [7]. Important deviations from the set point in metabolic steady states may ultimately cause damage to biomolecules and can modulate, and even disrupt, physiological redox signaling.

Embryonic development requires specific signaling events that regulate cell proliferation and differentiation to occur at the correct place and the correct time in order to build a healthy embryo. Signaling pathways are sensitive to perturbations of the endogenous redox state and are also susceptible to modulation by reactive species and antioxidant defenses, contributing to a spectrum of passive versus active effects that can affect redox signaling and redox stress [8].

Redox signaling plays a pivotal role in developmental processes, and it is largely regulated during embryogenesis. Disruption of redox signaling pathway alters the control of intracellular redox potential and causes redox stress through the generation of reactive oxygen species (ROS) [4]. These disruptions can include altered cell fate decisions that can lead to structural and functional changes in developing animals, including in specific tissues [9]. ROS and oxidative stress act as teratogenic agents, leading, during embryogenesis, to several structural changes in the developing fetus [8].

In addition to ROS, further important reactive species have notable impacts on redox biology and, consequently, on oxidative stress: reactive nitrogen species (RNS) [10], reactive sulfur species (RSS) [11], reactive carbonyl species (RCS), and reactive selenium species (RSeS) [12].

Enzymes such as superoxide dismutase (SOD), catalase, and glutathione peroxidase (GPx) are important in scavenging ROS and have been shown to increase 150% during the last 15% of gestation. There are three forms of SOD that have been identified: copper-zinc superoxide dismutase (Cu/ZnSOD) that is present primarily in the cytoplasm, manganese superoxide dismutase (MnSOD) in the mitochondria, and extracellular superoxide dismutase (EC-SOD) located in the extracellular spaces in adults but primarily intracellular in newborns. The only known function of SOD is to convert extremely reactive superoxide radicals to hydrogen peroxide and water. Catalase, GPx, and glutathione reductase then convert hydrogen peroxide to water [13].

Antioxidant enzyme expression generally increases in most fetal compartments throughout the progression of pregnancy. Qanungo and Mukherjea found that SOD, catalase, GPx, and glutathione reductase activities increased with gestational age, as evidence of lipid peroxidation decreased in human placental and fetal tissues [14].

Development of the antioxidant system during fetal life must also include redox signaling in the maintenance of pregnancy through uterine-placental-fetal interactions [15].

There is evidence of regulation of antioxidant enzymes in the context of local nitric oxide (NO) generation via nitric oxide syntheses and downstream NO-dependent signaling in the placenta, critically important to normal vascular development.

In this review, we present data concerning redox signaling in developmental processes and discuss the role of oxidative stress during pregnancy and embryogenesis. Models of congenital malformations in which redox modulations affect the development and function of the system are also shown.

## 2. Redox Signaling and Oxidative Stress during Embryonic Development

Intracellular ROS are important factors in signaling mechanism, as they modulate physiological processes during embryogenesis. Besides, cellular proliferation, differentiation, and apoptosis are often driven by specific redox signals.

Intracellular superoxide (O_2_-) is mainly produced by the oxidation of NADPH by NAPH oxidase enzymes (NOXs) or by electron leak from aerobic respiration in the mitochondria. Superoxide is then quickly converted into hydrogen peroxide (H_2_O_2_) by superoxide dismutases (SODs). H_2_O_2_ may either oxidize cysteine residues on proteins to initiate redox biology, or it may be converted to H_2_O by cellular antioxidant proteins, such as peroxiredoxins (PRx), glutathione peroxidase (GPx), and catalase (CAT). When H_2_O_2_ levels raise significantly, hydroxyl radicals (OH) form via reactions with metal cations (Fe^2+^) and irreversibly damage cellular macromolecules [5].

Hence, the homeostasis of intracellular oxidizing and reducing equivalents is modulated through a fine balance between antioxidant systems, enzymes, and metabolic processes to permit the normal cellular function that occurs when cell signaling is maintained and cellular viability is preserved [6].

Under normal physiological conditions, ROS are quickly destroyed by the antioxidant defense system. Free radical-mediated cellular damage may occur in case of genetic deficiency of free radical scavenging enzyme activity. An imbalance between diminished host antioxidant defenses and increased formation of free radicals (FRs) causes oxidative stress.

An increased production of ROS during organogenesis, period in which cells continue to differentiate, disrupts critical signaling events causing structural abnormalities, loss of cellular function, or spontaneous abortion of the developing fetus [7].

Different conditions may produce abundant ROS in human tissues, resulting in a state of oxidative stress for both the mother and the developing fetus. Although the relationship between oxidative stress and congenital malformations is not clear and need further investigations, experimental studies in animal models have shown that oxidative stress might play a significant causal role in birth defects.

For instance, Long and colleagues investigated the relationship between the toxicological effects of bacterial component LPS via oxidative stress and pulmonary dysplasia in chick embryos. The FGF and Wnt signaling pathways are considered to control lung development. GATA binding protein 6 (GATA-6) is a member of the zinc finger GATA protein family that presumably plays a key role in maintaining the balance between the proliferation and differentiation of pulmonary epithelial progenitor cells during lung development through modulating Wnt signaling. Long and colleagues demonstrated that LPS could induce the oxidative stress, which subsequently led to altered embryonic lung development. Specifically, LPS induced an intracellular ROS production enhancement, which was partially blunted by the addition of vitamin C to the culture medium. LPS significantly inhibited GATA-6 expression. However, GATA-6 was partially restored by vitamin C. Moreover, LPS induced downregulation of SP-C, ABCA3, and GATA-6 expressions, which again could be restored by vitamin C [16].

Over the course of development, the delicate balance between oxidants and antioxidants can be disrupted by various factors (e.g., thalidomide, phenytoin, ethanol, and maternal diabetes) that induce ROS production and lead to oxidative stress. Many investigators have evaluated the effects of antioxidants on embryonic development. In general, antioxidants reduce the abundance of highly reactive ROS by becoming radicalized themselves. The most important, glutathione, exists in a couple of its oxidized (GSSG) and reduced (GSH) forms. Another significant group of antioxidants are selenium and selenoproteins, such as thioredoxin, GSH peroxidase (GPX4), and selenoprotein W. Lipoic acid is a potent natural antioxidant. Mice deficient in lipoic acid are retarded in their development and die early with a lack of organization and smaller size. The heterozygotes have significantly reduced erythrocyte GSH levels and lower antioxidant capacity.

The enzyme G6PD is also very important to oxidative stress. It is a developmentally critical enzyme that protects the embryo from endogenous and xenobiotic-initiated oxidative stress and DNA damage. In fact, G6PD-deficient dams have higher embryonic oxidation and more fetal death and birth defects than their wild-type counterparts.

Other antioxidants, including vitamin C, are vital to the fetoplacental unit, so that in cryopreserved embryos, addition of ascorbate reduced the levels of hydrogen peroxide, increased the rate of metabolism, and enhanced inner cell mass development [17].

A better understanding of the mechanisms behind the relation between oxidative stress and congenital malformations may be important from both diagnostic and therapeutic perspective, providing both early prenatal diagnostic tools and new possible preventive treatments with antioxidant administration during pregnancy to reduce any oxidative damage of abundant ROS during organogenesis.

Oxidative stability can be assessed using different markers such as antioxidant enzymes: glutathione peroxidase (PxG), superoxide dismutase (SOD), catalase (CAT), glutathione S-transferase (GST), vitamin C, vitamin A, vitamin E, glutathione (GSH), or by determining the total antioxidant capacity (TAC) and nitrite levels [8].

Oxidative stress may also affect the levels of PAPP-A and *β*-HCG, already used to assess the risk of chromosomal aberration in the first trimester [9].

## 3. Oxidative Stress and Down Syndrome

Plentiful evidence both from in vivo and in vitro studies and animal models have suggested that the pathogenesis of Down syndrome (DS), due to complete or partial trisomy of chromosome 21, might be linked to the effects of oxidative stress on early embryonic development [18].

Trisomy of chromosome 21 causes deregulation of gene/protein expression either in dosage-sensitive genes (gene dosage hypothesis) or in several other genes (amplified developmental instability hypothesis) [19]. Furthermore, additional environmental factors—such as increased production of ROS—could play a central role in determining phenotype severity and the wide clinical variability observed in DS patients [20].

Previous studies have been supposed that a chronic oxidative injury in the brain would act as a risk factor for abnormal brain development, the higher incidence of hyperactivity with attention deficits and Alzheimer disease clinical features of DS patients [21, 22].

Genes located on chromosome 21, overexpressed due to triplication of the chromosome, include copper-zinc superoxide dismutase (SOD1), amyloid precursor protein (APP), transcription repressor BACH1 genes and other several genes with a role in ROS metabolism [23].

SOD1 gene encodes for the enzyme catalyzing the conversion of superoxide anion (O_2_^·^−) into the reactive oxygen species (ROS) hydrogen peroxide (H_2_O_2_) converted into water (H_2_O) and molecular oxygen (O_2_) by catalase (CAT) and glutathione peroxidase (GPX). The triplication of chromosome 21 causes an excessive activity of SOD1 and an altered ratio of SOD-1/CAT and GPX, resulting in the accumulation of endogenous H_2_O_2_ and/or its conversion products (hydroxyl radical) with cellular oxidative damage [24]. Interestingly, in several cells and tissues of DS patients, including erythrocytes, B- and T-lymphocytes, and fibroblasts, SOD1 levels are 50% higher than normal, and in all DS tissues there was an altered SOD1/GPX activity ratio [25]. A decreased expression of peroxiredoxin 2, an antioxidant enzyme that detoxifies hydrogen peroxide, was also detected in DS fetal brain [26]. These conditions make neurons of DS patients more sensitive to ROS attack and prone to apoptosis and degeneration [27].

It has also been reported both an increase of intracellular ROS and elevated levels of lipid peroxidation in primary human DS cortical cultures established from cerebral cortex of 16–19 weeks gestation. These evidences suggest that increased generation of ROS in fetal DS neurons leading to neuronal apoptosis may contribute to abnormal brain development and mental retardation and predispose to the early onset of Alzheimer's disease in DS as well [28].

Moreover, the analysis of oxidative stress biomarkers including enzymatic antioxidant defenses (CAT, SOD, and GPX) and oxidative damage antioxidants (protein carbonyls levels and lipoperoxidation), all measured spectrophotometrically in whole blood of 20 DS patients and 18 healthy controls, showed an increase in the SOD and CAT activities and a decrease in protein carbonyls levels in DS individuals, revealing a systemic prooxidant status in the blood of DS patients. Finally, in a cross-sectional study, total SOD activity in plasma from 36 DS children measured by spectrophotometric methods was found increased when compared with 40 healthy controls [29].

Trisomy 21 also causes overexpression of BACH1 gene, a basic leucine zipper protein belonging to the cap'n'collar (CNC) family. BACH1 is a transcription repressor that binds the antioxidant response elements of DNA (AREs) and suppresses the expression of specific genes/proteins controlled by ARE such as quinone oxidoreductase-1 (NQO1), glutathione S-transferase (GST), glutamate-cysteine ligase (GCL), and heme oxygenase-1 (HO-1). In oxidative stress conditions, the function of BACH1 is suppressed thus promoting the expression of these genes involved in the cell stress response. It was supposed that BACH1 overexpression might promote oxidative stress blocking the expression of oxidative stress-responsive and antioxidant genes. Increased total BACH1 protein levels were found into frontal cortex tissue from 16 DS individuals coupled with reduced induction of brain HO-1 compared to healthy individuals, suggesting that BACH1 overexpression in DS leads to the repression of HO-1 transcription and may contribute to the increased OS found in DS [30].

In addition, the overexpression of the amyloid precursor protein (APP) gene, located on chromosome 21, in DS patients causes an increased production of amyloid beta-peptide (A*β*) that is considered to be the most important pathogenic molecule in Alzheimer's disease representing the core protein of neuritic plaques. A*β* accumulates in the brains of DS individuals as early as 8–12 years of age, and this accumulation increases during the lifespan resulting in Alzheimer's disease-like neuropathology found in all DS individuals over 40 years of age. Soluble forms of A*β* generated from APP commonly end at C-terminal residue 40 (A*β*40) or 42 (A*β*42). Plasma concentrations of both A*β*40 and A*β*42, quantitated by sandwich ELISA from 35 DS children and adolescents, were reported significantly higher in DS patients than in controls and the ratio of A*β*42/A*β*40 was lower in DS than in controls [31]. This A*β*-peptide overexpression leads to accumulation into neuritic plaque inducing neuronal loss and cognitive dysfunction and could be associated with ROS production and oxidative stress [32].

Moreover, the overexpression of APP may also induce mitochondrial dysfunction independently from aberrant A*β* deposition, thus aggravating oxidative stress conditions [33].

Furthermore, there is another candidate gene for oxidative stress in DS patients encoding for the enzyme carbonyl reductase (CBR) that normally detoxifies the cytotoxic metabolic intermediates carbonyls catalyzing the reduction of free carbonyls compounds to their corresponding alcohols. Carbonyls are cytotoxic metabolic intermediates that are detoxified by either oxidation catalyzed by aldehyde dehydrogenase (ALDH) or by reduction to alcohols by CBR and/or alcohol dehydrogenase (ADH). Of note, increased levels of CBR protein have been shown in different brain regions of DS patients [34]. Thus, increased levels of CBR could be considered as a marker of oxidative stress, due to its role in detoxification of carbonyls produced by oxidative stress-dependent increases in SOD1 activity.

A systemic prooxidant status in DS individuals has been confirmed in various studies that demonstrated an increased activity of some important antioxidant enzymes (SOD1, CAT, and GR) together with decreased glutathione (GSH) levels in DS whole blood and higher levels of biomarkers of oxidative damage, such as protein carbonyls, malondialdehyde (MDA), allantoin, or 8-hydroxydeoxyguanosine than in controls [35].

Finally, the prooxidant condition in DS patients may be linked to reduced activity of complex I in the respiratory electron transport chain in the mitochondria associated with an increase in cellular ROS [36]. The oligonucleotide microarrays analysis of the expression profile of several genes located on chromosome 21 in 10 samples from cardiac tissue obtained from DS fetuses at 18–22 weeks of gestation after therapeutic abortion revealed a downregulation of genes encoding mitochondrial enzymes and upregulation of genes encoding extracellular matrix proteins. These results show that dosage-dependent upregulation of chromosome 21 genes alters the function of genes involved in mitochondrial function as well as the extracellular matrix organization of the fetal heart of DS patients [37].

In an effort to better understand the role of oxidative stress in DS, a set of oxidative biomarkers were evaluated in amniotic fluid collected from ten women undergoing amniocentesis and carrying confirmed DS fetuses compared with ten women carrying normal fetuses in a retrospective matched case control study [38]. Increased levels of circulating oxidative stress biomarkers were found. Particularly, protein carbonyls and HNE-protein adducts, both evaluated by slot-blot analysis, were found significantly increased in AF from women carrying DS fetuses, suggesting an improving of protein oxidation and lipid peroxidation pathways even at the fetal stage in DS. Glutathione assay results showed a reduction of total glutathione and an increase of GSSG levels with lower Trx levels in DS AF with respect to controls, confirming a loss of thiol-disulfide reductive systems. Furthermore, three heat shock proteins (HSP 70, heat shock protein 70; Grp 78 glucose regulated protein 78; and HO-1, heme oxygenase 1), acting through a cytoprotective mechanism under oxidative stress conditions, evaluated by western blot experiments, were found to be upregulated in DS AF.

It is clear from these data that DS fetuses are exposed to oxidative stress early in pregnancy with consequent damage of many fetal organs and tissues [39].

In conclusion, it has been suggested that trisomy of chromosome 21 causes stress oxidative conditions and oxidative injury early in embryogenesis altering gene/protein expression and particularly inducing overexpression of SOD1 and also reduction of antioxidant enzymes. Moreover, the overproduction of A*β* also affects redox imbalance and could exacerbate oxidative damage into the brain.

Table 1 summarizes the evidence discussed above.

Based on these findings, the administration of antioxidant nutrients could have a role in ameliorating the clinical pattern of DS patients. In previous studies on the effects of antioxidant elements, controversial results were obtained. In a 2-year randomized, double-blind, and placebo-controlled trial with daily oral antioxidant supplementation in DS patients and dementia (900 IU of alpha-tocopherol, 200 mg of ascorbic acid, and 600 mg of alpha-lipoic acid), it was demonstrated that the supplementation was safe and well tolerated but not associated with any stabilization or improvement in the cognitive function [40].

Further studies are needed in order to elucidate the relationship between oxidative stress and DS clinical expression to identify clinical biomarkers of early oxidative stress and damage and to find any possible therapeutic agents.

## 4. Oxidative Stress and Heart Malformation

The incidence of congenital heart defects (CHDs) varies from 4/1000 to 50/1000 live births [41].

The embryonal heart tube is composed of myocardium and an inner lining of endocardial cells separated by an extensive extracellular matrix the so-called cardiac jelly. The formation of cardiac cushions is a complex event under the direction of specific signaling pathways.

Nowadays, despite there are some progresses in understanding the genetics of heart defects, only 15% of CHDs can be attributed to a genetic cause. All other cases result from a complex interaction between genetic susceptibility and environmental factors (maternal cocaine and alcohol intake, cigarette smoking, exposure to industrial chemicals, viral infections, and so on) whose common embryotoxic effect might be related to excessive production of reactive oxygen species and to reduced antioxidant-defense mechanisms [42].

Despite the role of ROS in cardiovascular diseases (CVD) is well documented [43, 44], there are only few reports concerning the role of ROS in children with congenital heart defects (CHD) [45].

In a study by Ercan and colleagues, the relationship between congenital heart diseases and oxidative stress in children with cyanotic and acyanotic congenital heart diseases was investigated. The authors concluded that the oxidant and antioxidant values of the cyanotic patients were significantly higher than the acyanotic and control groups. So, they have speculated that due to the underlying anatomical defect, hypoxia develops and increases both the free oxygen radicals and the antioxidant substances for compensation afterwards [46].

Increased oxidative stress and reduced antioxidant capacity might lead to CHDs, through ROS production, which affect many intra- and intercellular signaling pathways [47, 48].

Furthermore, recent evidences in humans, i.e., mothers of offspring with congenital heart disease, have shown elevated homocysteine level related to low folate and/or vitamin B12 levels thus supporting that folic acid pathway alteration may exert an indirect embryotoxic effect by increasing oxidative stress. The metabolic pathway from homocysteine to glutathione is referred to as the transsulfuration pathway. Approximately 50% of homocysteine generated from methionine is metabolized to cystathionine. This is an irreversible reaction that permanently removes homocysteine from the methionine cycle for the synthesis of cysteine and glutathione. Elevated homocysteine is associated with alterations in the transsulfuration pathway that lead to greater oxidative stress [49]. In a previous publication, evidence of impairment in remethylation of homocysteine was shown by lower methionine and S-adenosylmethionine concentrations and higher S-adenosylhomocysteine concentrations among women with CHD-affected pregnancies [50]. Current findings indicate that the higher homocysteine observed among women with CHD affected pregnancies may extend beyond impairments in remethylation of homocysteine to impairments in the transsulfuration of homocysteine. Specifically, in comparison to controls, cases with CHD-affected pregnancies had significantly lower concentrations of vitamin B-6, GluCys, and GSH and significantly higher concentrations of GSSG [50].

Experimental models have suggested that, in addition to evidence of a direct teratogenic effect, elevated homocysteine may have an indirect embryotoxic effect by increasing oxidative stress through excessive production of reactive oxygen species and by decreasing the glutathione-dependent antioxidant-defense mechanism. Hobbs and colleagues indicate that higher homocysteine observed among women with CHD-affected pregnancies may extend beyond impairments in remethylation of homocysteine to impairments in the transsulfuration of homocysteine. Specifically, in comparison to controls, cases with CHD-affected pregnancies had significantly lower concentrations of vitamin B-6, glutamylcysteine (GluCy), and reduced glutathione (GSH) and significantly higher concentrations of oxidized glutathione (GSSG).

Role of oxidative stress for congenital cardiovascular malformations is well studied in maternal diabetes [51]. Congenital heart disease occurs in 5% of infants of diabetic mothers.

In fact, diabetic pregnancy is considered an independent risk factor for major embryonic malformations, and cardiac outflow tract defects are among the most frequent alterations observed in epidemiological studies [52]. The highest relative risk for major cardiovascular defects occurs if the mother has gestational diabetes and develops insulin resistance in the 3^rd^ trimester.

Studies on rats have shown that hyperglycemia in the embryo induces production of reactive oxygen species that, together with a reduced ability of fetal cells to activate antioxidant defense mechanisms, mediate adverse effects on cardiac neural crest migration and cardiac outflow tract septation through proapoptotic signaling [53, 54].

In humans, oxidative stress markers have been evaluated in the cord blood of newborns delivered by mothers with diabetes, suggesting that hyperglycemia induces oxidative stress [55]. However, glucose itself is not a mutagen; instead, it may exert a teratogenic effect via a signaling pathway regulating insulin sensitivity. Insulin sensitivity is thought to be involved in the pathophysiology of both type 1 and type 2 diabetes mellitus and insulin, and related signaling pathways are also key mediators of embryogenesis and early development [56].

Glucose may also affect gene expression in embryonic development via epigenetic changes (histone acetylation and microRNA expression) [57].

The alternative that offspring CHD reflects maternally inherited genetic or epigenetic variations that confer risk of both diabetes mellitus and cardiac abnormalities is less likely, because the risk of maternal diabetes mellitus subsequent to birth of a child with CHD was only modestly increased. Conotruncal defect risk increased in the offspring of diabetic women consistent with experimental study findings that hyperglycemia in early pregnancy affects regulatory gene expression in the embryo, leading to cardiac neural crest cell death and increased CHD risk, particularly for conotruncal and outflow tract abnormalities.

Detailed mechanistic studies will be required to define the role of glucose sensitivity in cells from the neural crest and anterior second heart field during cardiac development. Maternal diabetes mellitus was also associated with the entire spectrum of CHD phenotypes. The nonspecific nature of the association suggests that hyperglycemia in early pregnancy may not only influence specific sequences in cardiac development but affects cardiac development in general or exerts its detrimental effect before formation of the primitive heart tube, with subsequent early and late consequences for fetal cardiac development.

Studies in rats have shown that oxidative stress during pregnancy can be reduced by using vitamin E and Vitamin C as antioxidants [58, 59], thereby supporting that ROS are involved in the embryonic dysmorphogenesis of diabetic pregnancy.

Furthermore, recent evidences in humans, i.e., mothers of offspring with congenital heart disease, have shown elevated homocysteine level related to low folate and/or vitamin B12 levels [50, 60], thus supporting that folic acid pathway alteration may exert an indirect embryotoxic effect by increasing oxidative stress [61].

Despite some human studies have shown that women using multivitamin supplements and folic acid during the periconceptional period had a lower risk of having babies with congenital heart defects, there are still concerns about the role of multivitamin supplements in reducing embryotoxic effect and risk for CHD in humans [62, 63].

Recent studies have also shown that ROS overproduction and/or imbalance in the antioxidant system could lead to pulmonary hypertension in cases of CHD associated with increased pulmonary blood flow in lamb [64] and rodent experimental model of congenital diaphragmatic hernia [65] through a disordered process of vascular remodeling leading to smooth muscle cell hyperplasia, hypercontractility, and endothelial dysfunction [66].

Further studies are needed to identify the key factors in the development of CHDs in order to develop and implement effective primary prevention program with preconceptional screening and development of nutritional intervention.

Table 2 summarizes the factors that favour or prevent CDH via oxidative stress.

## 5. Role of Oxidative Stress in Neural Tube Defect

**Figure 1:**
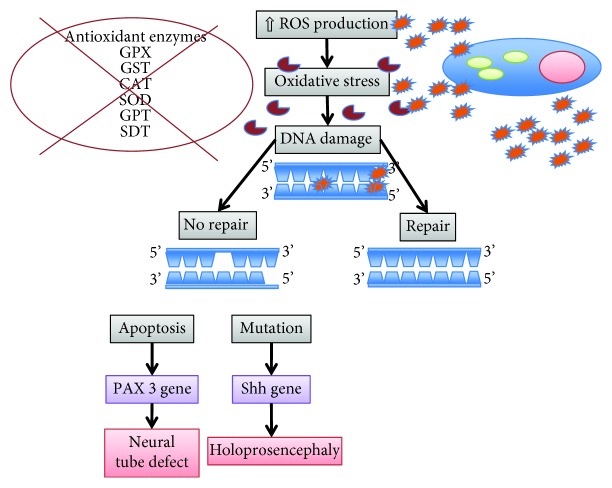
Oxidative stress and CNS malformations.

The human brain is particularly vulnerable to the damaging effects of reactive oxygen intermediates due to both its complexity and the long period of development (Figure 1).

Embryonic and fetal brain tissues are especially susceptible to peroxidative injury due to the fact that their membranes are rich in easily oxidable polyunsaturated fatty acid side chains [67].

Several studies indicate that antioxidant enzymes and molecules exhibit extremely low activities in fetal tissues, especially the brain [25, 68]. Cim and colleagues demonstrated higher levels of MDA, indicating an increased oxidant status in amniotic fluid of pregnant women with fetal congenital malformations of the central nervous system [69]. Although this antioxidant defense system is adequate to protect brain development under normal conditions, it is easily overcome by ROS, resulting in neurological and morphological abnormalities of SNC [70].

Holoprosencephaly (HPE) is one of the most common birth defects and is characterized by midline defects of the brain, facial, and oral structures. In humans, it has been estimated that HPE affects 1 in every 5000–10,000 live births and 1 in every 200–250 miscarried fetuses [71]. Many cases of human HPE occur following fetal alcohol exposure or as a result of maternal diabetes both associated with elevated levels of reactive oxygen species. Studies on the teratogenic mechanism of ethanol-induced HPE in mice have showed that ethanol may impair Sonic hedgehog (Shh) gene expression by activation of protein kinase A (PKA), a potent endogenous negative regulator of Shh signaling during the development of the neural tube [72, 73].

Shh, produced by the axial mesendoderm, prechordal mesoderm (PME), and notochordal plate, acts as a crucial signal in mammalian brain and facial development, and Shh gene alterations are the most frequent causes of autosomal-dominant inherited HPE in humans and Shh2/2 mouse embryos exhibit severe HPE [74–76].

Ethanol-induced activation of PKA in the anterior PME results in a reduction of Shh expression and enhanced apoptosis of anterior PME cells causing the characteristic severe midline defects of HPE.

The inhibition of PME cells apoptosis by antioxidants, i.e., vitamin C and vitamin E, may protect from the teratogenic actions of ethanol.

Neural tube defects (NTDs) are a group of common and devastating congenital malformations that arise early in pregnancy due to the disturbance of normal neural tube closure. NTDs occur in about one in every 1000 established pregnancies worldwide [77], and it is estimated that over 323,000 births were affected with NTDs globally in 2001 [78].

The aetiology of NTD is thought to be heterogeneous, including genetic and environmental factors and their interactions [77, 79]. Factors that have been found to be associated with the risk of NTDs include insufficient folate [80] or multivitamin [81] intake, pregestational and gestational diabetes [82], pesticides [83], and antiepileptic drugs [84]. However, the proportion of NTD cases that can be attributed to known risk factors is lower than one-third [85]. Studies to delineate the mechanism underlying maternal diabetic embryopathy have demonstrated that oxidative stress is a major contributor in NTD formation [86–89]. Excess apoptosis may be one of the mechanisms by which oxidative stress induces malformations. Apoptosis occurs at various developmental stages as a homeostatic mechanism to maintain cell populations in tissues [90]. During the formation of the neural tube, apoptosis appears to be dispensable; however, excessive apoptosis could potentially result in NTDs by causing insufficient cells to be present in the fusing neural folds or by disrupting the physical continuity of the dorsal midline [77, 90]. Growing evidence indicates that oxidative stress can stimulate apoptosis, which may lead to insufficient cell numbers to participate in folding and fusion of neural walls of the neural tube [90, 91].

Moreover, oxidative stress induces DNA damage and defects in DNA repair mechanisms. Single- and double-stranded fractures, base modifications (base participation, rearrangement in some cases), and nucleoside damage may occur in DNA. There may also be crosslinking between DNA and protein depending on oxidative damage [92, 93]. Early embryonic development is vulnerable to oxidative stress because of the immaturity of free radical scavenging mechanisms [69]. The paired box 3 (*Pax3*) gene plays a major role in the development of neuroepithelium of embryos. In the absence of *Pax3*, neural tube defects occur [94]. Oxidative stress occurring before *Pax3* expression leads to an increased risk of neural tube defects and diminished gene expression [95].

Myelomeningocele (MM) is a common congenital malformation that occurs when the embryonic neural tube fails to close properly during early embryogenesis. Common pathogenetic mechanisms for MM include folate deficiency, genetic susceptibility, environmental factors, in utero drug exposure, and biochemical factors [95–100].

Numerous reports have described free radical-mediated congenital defects [86–89]. Kao and colleagues supported the role of folate in modulating intracellular oxidative stress and suggest an additional mechanism for the etiology of folate deficit-associated MM [101]. Indeed, there is a direct relationship between antioxidant enzymes and the development of MM. GPX, GST, and SOD enzymes are the most important protective systems in humans for neural tube defects. An impaired responsiveness of the antioxidant enzymes, such as CAT, SOD, GPT, and SDT that play an active role in the detoxification of hydrogen peroxidase, has crucial effects in oxygen-induced embryopathy and might result in MM [102, 103].

In addition, SOD is involved in FR-mediated neurological diseases and acting a fundamental role in modulating reactive oxygen species toxicity [104]. In tissues lacking significant catalase activity, detoxification of hydrogen peroxidase becomes critically dependent on GPX. In the study of Graf and colleagues, enzyme activity was abnormal in MM children compared to a control group underlying as deficiencies of enzyme are directly linked to neural tube defects [105].

Arslan and colleagues found that malondialdehyde (MDA), an oxidative damage marker, and a lower activity of erythrocyte carbonic anhydrase, an antioxidant enzyme which regulates the acid-base homeostasis, differ in newborns with MM and in their mothers compared to healthy newborns and their mothers [106].

This study suggests that the finding of low antioxidant enzyme activities in addition to ultrasound and maternal serum alpha fetoprotein may be an index of suspicion of neural tube defect.

In pregnant women with high risk, antioxidant enzymes administration together with folic acid may be an opportunity to reduce the incidence of neural tube defect.

## 6. Conclusion

Pregnancy is a state of oxidative stress as a consequence of high metabolic activity in the fetoplacental compartment. Fetal tissues are especially sensitive to oxidative damage because of the rapidly growing nature of their cells, which makes them vulnerable to the harmful effects of free radicals.

Despite reactive oxygen species and free radicals, in the presence of a good antioxidant capacity, are important for developing embryos, promoting and controlling cellular fate, and playing a crucial role in normal development through cellular signaling, when overproduced, in the absence of a parallel increase in antioxidative activity, resulted in a wide range of biological toxic effects.

Due to the rapidly growing nature of their cells, fetal tissues are especially sensitive to oxidative damage that lead to lipid, protein, and polysaccharides oxidation and DNA damage with disruption of apoptosis processes that, during organogenesis, are highly needed in an appropriate location and temporal pattern.

Thus, the increase of oxidative stress, together with the impaired antioxidant activity, is clearly related to the induction of fetal malformations.

There are still gaps in our knowledge in the role of oxidative injury in the activation of complex array of genes involved in different biological processes of fetal structure such as inflammation, coagulation, fibrinolysis, cell cycle, cell adhesion, and signal transduction. Future studies addressing the role of oxidative stress in this field are encouraged.

Moreover, there are few published studies evaluating oxidative stress biomarkers and management of oxidative stress with antioxidants therapeutic approaches.

Oxidative stress is widely implicated in failed reproductive performance, including infertility, miscarriage, diabetes-related congenital malformations, and preeclampsia. Poston et al. have focused on the role of free radicals and antioxidant capacity in preeclampsia. By measuring markers of lipid peroxidation and antioxidant capacity, they demonstrated the role of oxidative stress in this disorder [107].

Recent studies suggest that ischemia-reperfusion in the placenta as well as endoplasmic reticulum stress in the placenta may contribute to oxidative stress in trophoblasts. The recognition of oxidative stress in the placenta and the maternal circulation has led to evaluate the potential benefit of prophylactic antioxidant supplementation in women with a known risk of preeclampsia, particularly with an early supplementation with vitamins C and E [108]. However, until now, trials have shown no evidence that these supplements can prevent preeclampsia, but it is important to underline that no RCT has yet addressed prophylaxis over the periconceptual period.

Other potential approaches include the use of supplements in the preconceptual period, selenium supplements, antiperoxynitrite strategies, and statins [109, 110].

In clinical practice, early markers of oxidative stress might reveal that gravidic prophylactic use of antioxidants could help to prevent or at least reduce oxidative stress-related malformations in fetuses. Anyway, maternal antioxidant supplementation during pregnancy is important for protecting newborns against oxidative DNA damage.

Potential therapies for ROS-induced disease include both enzymatic and nonenzymatic antioxidant preparations. Supplementation with enzymatic and/or nonenzymatic antioxidants might have beneficial effects in decreasing injury from excess production of ROS, particularly in disorders such as bronchopulmonary dysplasia, retinopathy of prematurity, periventricular leukomalacia, and necrotizing enterocolitis in preterm newborns who are especially susceptible to ROS-induced damage because of inadequate antioxidant stores at birth, as well as impaired upregulation in response to oxidant stress [13]. Nonenzymatic proteins (transferrin, ferritin, and ceruloplasmin), enzymes (superoxide dismutases, catalase, and glutathione peroxidase), oxidizable molecules (glutathione, vitamins E, A, C, carotenoids, and flavonoids), and trace elements (copper, zinc, and selenium) all play a role in maintaining a delicate balance between ROS production and oxidant damage to tissues and organs [111, 112].

More research is required to determine the beneficial effects of supplemental antioxidant therapy. There are multiple potential therapeutic antioxidants currently under investigation that could benefit newborns, particularly premature infants. For example, one protein under investigation is Pon3 that was shown in laboratory studies to have antioxidant properties and to be upregulated in rat, sheep, and human cord blood late in gestation [113]. Other clinical trials include supplementation of preterm infants with lactoferrin and cysteine, examination of concentrations of beta-carotene, lutein, and lycopene in preterm infants fed formulas with mixed carotenoids and the effects on the developing eye, early administration of human erythropoietin in very preterm infants, NAC administration to women with intra-amniotic infection and/or inflammation, early enteral administration of vitamin E to extremely premature infants, and multiple trials involving inhaled nitric oxide. The results from these trials may change the way we treat many common neonatal conditions.

Caution must be taken since ROS are critical second messengers in various cell signaling pathways that control normal cellular functions, but strategies that maintain normal antioxidant balance may be beneficial to the newborns. New studies should more extensively investigate the diagnostic and therapeutic value of various oxidative stress biomarkers and antioxidants to reduce oxidative tissue injury to developing newborns.

## Figures and Tables

**Table 1:** Summary of the markers investigating in relation to oxidative damage in Down syndrome.

Marker	DS cell/tissue	Level
SOD1	Erythrocytes	Increased
	B- and T-lymphocytes	Increased
	Fibroblasts	Increased
	Whole blood	Increased activity

Peroxiredoxin 2	Fetal brain	Decreased

CAT	Whole blood	Increased activity

Protein carbonyls	Whole blood	Increased
	Amniotic fluid	Increased

Total Glutathione GSSG	Amniotic fluid	Increased
		Increased
Trx		
		Decreased

ROS	Primary human cortical cell cultures	Increased

Lipid peroxidation	Primary human cortical cell cultures	Increased

BACH1 protein	Frontal cortex tissue	Increased

A*β*40 and A*β*42	Plasma	Increased

CRB	Brain	Increased

GSH	Whole blood	Decreased

HNE-protein adducts	Amniotic fluid	Increased

HSP 70 Grp 78 HO-1	Amniotic fluid	Increased

**Table 2:** Factors that favour or prevent CHD via oxidative stress.

Favouring	Preventing
(i) Maternal diabetes	(i) Vitamin E
(ii) Hyperhomocysteine	(ii) Vitamin C
	(iii) Folic acid
	(iv) Vitamin B12
